# SiO*_x_*-Based Anode Materials with High Si Content Achieved Through Uniform Nano-Si Dispersion for Li-Ion Batteries

**DOI:** 10.3390/ma18143272

**Published:** 2025-07-11

**Authors:** Seunghyeok Jang, Jae-Hun Kim

**Affiliations:** School of Materials Science and Engineering, Kookmin University, Seoul 02707, Republic of Korea; jangss1124@kookmin.ac.kr

**Keywords:** Si based material, Si suboxide composite, high capacity, anode material, Li-ion battery

## Abstract

Silicon alloy-based materials are widely studied as high-capacity anode materials to replace commercial graphite in lithium-ion batteries (LIBs). Among these, silicon suboxide (SiO*_x_*) offers superior cycling performance compared to pure Si-based materials. However, achieving a high initial Coulombic efficiency (ICE) remains a key challenge. To address this, previous studies have explored Si_x_O composites (x ≈ 1, 2), where nano-Si is uniformly dispersed within a Si suboxide matrix to enhance ICE. While this approach improves reversible capacity and ICE compared to conventional SiO, it still falls short of the capacity achieved with pure Si. This study employs a high-energy mechanical milling approach with increased Si content to achieve higher reversible capacity and further enhance the ICE while also examining the effects of trace oxygen uniformly distributed within the Si suboxide matrix. Structural characterization via X-ray diffraction, Raman spectroscopy, and electron microscopy confirm that Si crystallites (<10 nm) are homogeneously embedded within the SiO*_x_* matrix, reducing crystalline Si size and inducing partial amorphization. Electrochemical analysis demonstrates an ICE of 89% and a reversible capacity of 2558 mAh g^−1^, indicating significant performance improvements. Furthermore, carbon incorporation enhances cycling stability, underscoring the material’s potential for commercial applications.

## 1. Introduction

Widespread reliance on fossil fuels presents significant challenges, including resource depletion, environmental pollution, and increasing difficulties in exploration [1]. As a result, extensive efforts are underway to develop alternative energy sources. Countries such as Germany, France, and the UK have announced plans to phase out internal combustion engine vehicles, contributing to a projected rise in the global electric vehicle market share [2]. Alongside this shift, the demand for high-energy-density power sources is growing rapidly, driven by the increasing use of electronic devices. Among these power sources, lithium-ion batteries (LIBs) are widely recognized for their cost-effectiveness, long lifespan, and high energy density, making them a cornerstone of next-generation energy solutions. However, the current performance of LIBs remains insufficient to meet escalating energy demands, necessitating advancements in battery technology to achieve higher energy densities [3,4,5].

Graphite-based materials are the dominant commercial anodes in LIBs. Graphite accommodates Li ions (Li^+^) through intercalation, offering excellent lifespan characteristics and high initial Coulombic efficiency (ICE). However, its reversible capacity is inherently limited to 372 mAh g^−1^ due to constraints imposed by its crystal structure, making it unsuitable for high-capacity energy storage applications. Li alloy-based materials are considered promising alternatives, with Si emerging as one of the most attractive candidates due to its exceptionally high theoretical capacity (~10 times that of graphite, 3580 mAh g^−1^ for the Li_3.75_Si phase) [6,7,8,9]. Despite this advantage, Si undergoes substantial volume expansion (~400%) during charge and discharge cycles, leading to mechanical degradation. This results in the electrical isolation of Si particles from the current collector and the formation of a solid electrolyte interphase (SEI) layer due to side reactions between Si and the electrolyte. These issues reduce Coulombic efficiency and cause rapid capacity degradation [10,11,12].

To mitigate these challenges, research has focused on carbon composites and nanotechnology-based approaches. Various structural designs, including Si nanowires, nanotubes, and core–shell architectures, have been explored to enhance the cycling performance of Si-based electrodes [13,14,15,16,17,18,19]. However, the high manufacturing costs and complexity of producing these nanostructures pose significant barriers to large-scale commercialization.

Silicon suboxides (SiO*_x_*, 0 < x < 2) have emerged as promising Si-based anode materials for LIBs, with silicon monoxide (SiO) being extensively studied [20,21,22,23]. Transmission electron microscopy (TEM) and X-ray diffraction (XRD) studies have shown that amorphous SiO contains nanocrystalline Si dispersed within a SiO*_x_* matrix [24,25]. Additionally, solid-state nuclear magnetic resonance and X-ray photoelectron spectroscopy analyses have revealed that SiO exhibits multiple oxidation states ranging from 0 to +4. The unique microstructure of nanocrystalline Si embedded in a Si suboxide matrix effectively mitigates volume expansion during lithiation and delithiation, leading to improved cycling stability compared to conventional Si-based materials [26,27,28]. Previous research has explored Si_x_O (x = 1, 2) composites, where nano-Si is uniformly dispersed within a Si suboxide matrix at Si:O molar ratios of 1:1 and 2:1, respectively. These materials have demonstrated enhanced capacity, cycling performance, and ICE compared to SiO [29,30].

This study aims to further improve reversible capacity and ICE by increasing the Si content in Si_x_O. The electrochemical effects of trace oxygen in the Si suboxide matrix were also investigated. The Si_5_O composite was synthesized using high-energy mechanical milling (HEMM). TEM analysis confirmed a microstructure in which nanocrystalline Si is uniformly dispersed within the Si suboxide matrix. XRD, Raman spectroscopy, and electrochemical analyses revealed that excessive dispersion of nanocrystalline Si led to partial amorphization due to the energy imparted by the milling process. The simply milled Si_5_O composite achieved a high reversible capacity of 2558 mAh g^−1^ and an ICE of 89%, demonstrating superior performance compared to previous studies due to the presence of uniformly dispersed trace oxygen. Additionally, subsequent carbon incorporation further enhanced cycling stability, underscoring the material’s potential for commercial applications. These findings highlight the promise of Si_5_O composites as high-performance anode materials for next-generation LIBs, offering a scalable approach to improving energy density and cycling stability.

## 2. Experimental

**Material synthesis**: The Si_x_O composites were synthesized via a one-step HEMM process. Si nanopowder (nano-Si, <100 nm, Sigma-Aldrich, St. Louis, MO, USA) and silicon monoxide (SiO, 325 mesh, Sigma-Aldrich) were used as precursor materials. These powders were mixed in molar ratios of 3:1, 4:1, 5:1, 7:1, and 10:1 inside a stainless-steel vessel. Stainless-steel balls (4 mm and 10 mm diameter) were added at a weight ratio of 20:1 relative to the total powder mass. The milling process was carried out in an argon-filled glove box using a laboratory-built mechanical mill (SPEX-type) at 800 rpm for 24 h, producing Si_x_O (x = 3, 4, 5, 7, and 10) composites. To enhance cycling performance, a carbon coating was applied to the Si_5_O composite through the carbonization of naphthalene. The composite was mixed with naphthalene and heated in a vertical furnace under an argon atmosphere. The temperature was increased at a ramp rate of 5 °C min^−1^ to 900 °C and held for 2 h. After cooling to room temperature, the final product was a carbon-coated Si_5_O@C composite.

**Material characterization**: The crystal structures of the synthesized materials were analyzed using X-ray diffraction (XRD, Rigaku ULTIMA IV, Tokyo, Japan) with Cu Kα radiation. Raman spectroscopy (LabRAM Soleil, Horiba Scientific, Irvine, CA, USA) was used to investigate changes in Si bonding. The structure of samples was further examined via Fourier-transform infrared spectroscopy (FT-IR, Nicolet iS50, Thermo Scientific, Waltham, MA, USA; resolution: 0.482 cm^−1^). X-ray photoelectron spectroscopy (XPS, ESCALAB 250, Thermo Scientific, Waltham, MA, USA) with Al Kα radiation was performed to further examine the chemical bonding states. The binding energy scale was calibrated using the C 1s peak at 284.8 eV as a reference. The microstructures of the Si_x_O (x = 3, 4, 5, 7, and 10) composites were characterized using field-emission scanning electron microscopy (FE-SEM, JSM-7410F, JEOL, Tokyo, Japan) and high-resolution transmission electron microscopy (HR-TEM, JEM-2100F, JEOL, Tokyo, Japan), operated at an accelerating voltage of 200 kV and equipped with energy-dispersive spectroscopy (EDS).

**Electrochemical measurements**: The electrode slurry was prepared by dissolving the active material, a conductive agent (Super P, Sigma-Aldrich), and a binder (polyacrylic acid, Sigma-Aldrich) in deionized water at a weight ratio of 7:1.5:1.5. This slurry was coated onto a copper foil, roll-pressed, and dried under vacuum at 80 °C for 12 h. The electrode was then cut into 12 mm diameter disks. Half-cell electrochemical tests were conducted using CR2032-type coin cells, which consisted of the working electrode, a Li foil counter/reference electrode, a porous polyethylene separator, and an electrolyte. The electrolyte was a 1 M LiPF_6_ solution in ethylene carbonate/diethyl carbonate (3:7 by volume, Enchem Co., Jecheon-si, Chungbuk, Republic of Korea) containing 10% fluoroethylene carbonate. Coin cells were assembled in an argon-filled glove box (Korea Kiyeon, Seoul, Republic of Korea) under controlled atmospheric conditions (O_2_ ≈ 0 ppm, H_2_O < 0.5 ppm). Galvanostatic charge/discharge tests were performed using a battery cycler (Basytec CTS-Lab, Baden-Württemberg, Germany) at various current densities within a voltage range of 0.01–2.0 V (vs. Li^+^/Li). The 1C rate was set to 1000 mA g^−1^ based on the capacity of the synthesized Si_x_O composites. To evaluate practical applicability, a blended electrode was prepared by mixing Si_5_O@C and graphite at a 1:9 weight ratio. This blended anode was fabricated using a mixture of the active material, carboxymethyl cellulose, and styrene–butadiene rubber in deionized water at a weight ratio of 96:2:2. Electrochemical impedance spectroscopy (EIS, VSP, BioLogic V11.43, Grenoble, France) measurements were conducted before cycling and after 30 cycles over a frequency range of 1 MHz to 0.1 Hz, with an applied AC amplitude of 5 mV.

## 3. Results and Discussion

**Figure 1:**
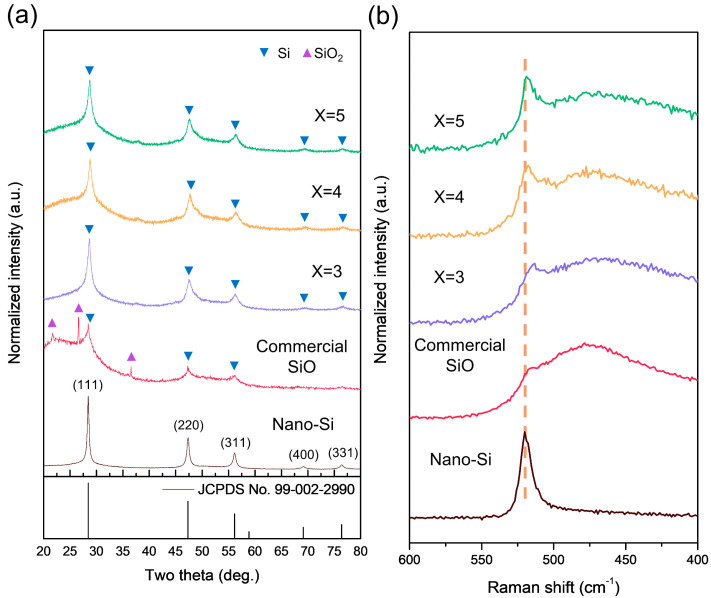
(**a**) XRD patterns and (**b**) Raman spectra of the composites for Si_x_O (x = 3, 4, and 5) and reference samples.

Figure 1a presents the XRD patterns of Si_x_O (x = 3, 4, and 5) composites synthesized via HEMM. The commercial SiO precursor exhibits broad peaks characteristic of its amorphous nature, along with SiO_2_ (quartz) and Si peaks, consistent with previous reports. These observations suggest that SiO is typically produced by the simultaneous evaporation of Si and SiO_2_ under vacuum at high temperatures (>1400 °C), resulting in residual unreacted Si and SiO_2_ [25,31].

The XRD patterns of the milled Si_x_O composites no longer show SiO peaks, apart from the Si peak, indicating successful composite formation. However, in composites with Si:O molar ratios of 7:1 and 10:1, FeSi_2_ peaks emerge, likely due to the reaction between unreacted nano-Si and the stainless-steel container during the HEMM process (Figure S1). Since FeSi_2_ is electrochemically inactive and reduces the reversible capacity [32], further analysis focuses on Si_x_O (x = 3, 4, and 5), where FeSi_2_ formation is absent. The Si_x_O (x = 3, 4, and 5) composites exhibit broad diffraction peaks in the 20–25° range. Compared to precursor nano-Si, the peaks of these composites are broader, suggesting that oxygen from SiO has reacted with Si to form SiO*_x_* or that the milling-induced mechanical energy has partially amorphized the crystalline nano-Si.

The Raman spectra of the milled samples are shown in Figure 1b. The nano-Si precursor (<100 nm) exhibits a sharp Si–Si vibrational peak at ~520 cm^−1^, indicative of crystalline Si. In contrast, commercial SiO displays a broad peak between 470 and 520 cm^−1^, as previously reported. For Si_x_O (x = 3, 4, and 5), the intensity of the Si–Si peak at ~520 cm^−1^ increases with higher Si content, and the peak broadens compared to nano-Si. This broadening is attributed to the presence of excess Si, some of which remains crystalline, while unreacted Si undergoes partial amorphization due to milling-induced energy. Additionally, oxygen from SiO reacts with Si during milling, forming amorphous SiO*_x_*, which contributes to the broad peak between 470 and 520 cm^−1^. The Raman results align with the XRD findings, confirming the coexistence of amorphous SiO*_x_* and partially amorphized Si [33]. Figure S2 presents the FT-IR spectra, which shows trends consistent with the Raman spectra.

FE-SEM images of the precursor nano-Si, SiO, and the synthesized Si_5_O composite are shown in Figure S3. The nano-Si precursor, with a particle size of <100 nm, exhibits the smallest dimensions among the materials. In contrast, SiO particles range from a few micrometers to several tens of micrometers and exhibit sharp morphologies. The Si_5_O composite synthesized via ball milling lacks a distinct shape due to the HEMM process, which crushes the powders, producing particles comparable in size to or smaller than SiO particles. Additionally, while SiO particles retain relatively angular shapes, the Si_5_O composite displays rounded edges.

**Figure 2:**
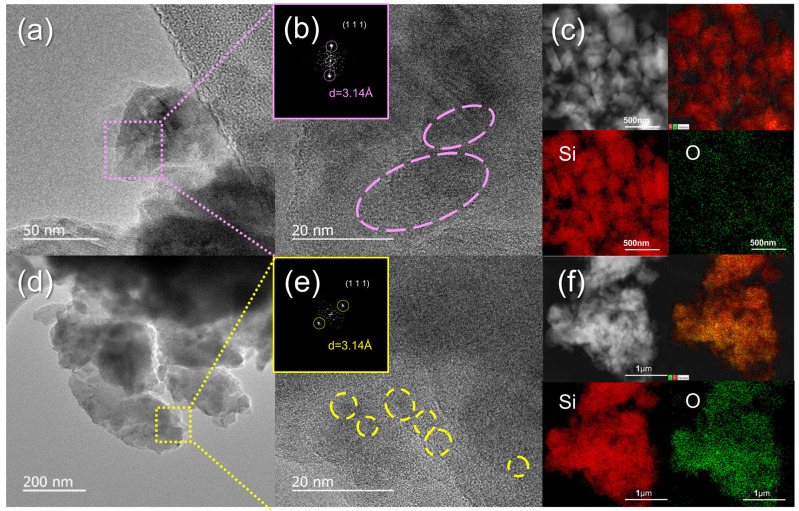
HR-TEM and EDS elemental mapping images of (**a**–**c**) nano-Si and (**d**–**f**) Si_5_O samples (inset: FFT patterns).

Figure 2 presents HR-TEM images and the EDS elemental mapping of precursor nano-Si and the Si_5_O composites. The HR-TEM images of nano-Si (Figure 2a,b) reveal Si nanocrystallites measuring 15–25 nm (Figure S4a). After milling, HR-TEM images of the Si_5_O composite (Figure 2d,e) show that nanocrystalline Si is embedded within an amorphous SiO*_x_* matrix, consistent with previous reports [34,35,36]. The milling process leads to a uniform distribution of Si crystallites within the SiO*_x_* matrix and reduces their size to 5–10 nm (Figure S4b). Since a nonuniform distribution and broad crystallite size range can hinder the accommodation of mechanical stress during volume changes, the spatially homogeneous distribution and narrow size range of Si crystallites in Si_5_O are expected to enhance cycling performance [29,30].

The insets of Figure 2b,e show the fast Fourier-transform (FFT) patterns of nano-Si and the Si_5_O composite, confirming the presence of Si nanocrystals within the amorphous SiO*_x_* matrix. A d-spacing of 3.14 Å is observed in the crystalline Si lattice, corresponding to the (111) reflection of the Si crystal structure. Additionally, EDS elemental mapping (Figure 2f) confirms the uniform distribution of Si and O in the Si_5_O composite.

**Figure 3:**
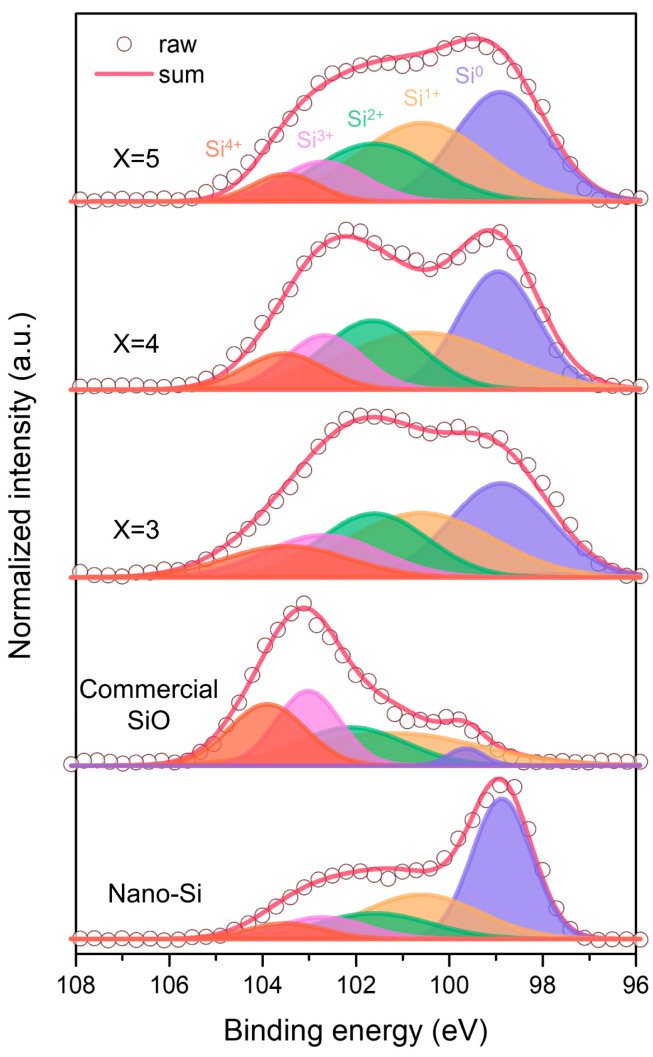
XPS core-level spectra of Si 2p for the Si_x_O composite (x = 3, 4, and 5) and reference samples.

Figure 3 presents the XPS core-level spectra of Si 2p for the Si_x_O (x = 3, 4, and 5) composite samples and the reference materials (as-received nano-Si and commercial SiO). The XPS signals were measured after surface cleaning via Ar ion sputtering for 300 s. The spectra reveal that Si exists in oxidation states ranging from 0 to +4. Each Si 2p spectrum was deconvoluted into peaks corresponding to Si^0^, Si^1+^, Si^2+^, Si^3+^, and Si^4+^ [25,26,34]. To clarify the distribution of oxidation states, the area ratios of the deconvoluted peaks were calculated and are listed in Table 1.

For nano-Si, the Si^0^ peak is the most dominant (42.9%), consistent with the presence of native oxide films on nanosized Si powders, which also contain oxidation states from +1 to +4. In contrast, the Si 2p spectrum of commercial SiO exhibits the smallest Si^0^ peak area (3.1%) and a relatively even distribution among Si^1+^ (28.4%), Si^2+^ (20.9%), Si^3+^ (24.0%), and Si^4+^ (23.6%). As the Si ratio increases in Si_x_O (x = 3, 4, and 5), the Si^0^ and Si^1+^ peaks become more prominent. After the HEMM process, the Si^0^ content in Si_x_O increases from 3.1% in SiO to 27.9%, 29.1%, and 31.6% for Si_3_O, Si_4_O, and Si_5_O, respectively. Similarly, the Si^1+^ content rises from 28.4% to 26.8%, 28.1%, and 31.1%. This trend is attributed to the higher Si content in these composites, which promotes the formation of Si suboxides, particularly Si^0^ and Si^1+^, due to the stoichiometric excess of Si (with Si:O molar ratios of 3:1, 4:1, and 5:1). Si^0^ and Si^1+^ are the active species that reversibly alloy with Li, thus directly contributing to the high specific capacity. In contrast, Si^2+^ to Si^4+^ species are electrochemically less active but serve as a mechanically robust buffer matrix, accommodating volume changes during cycling and enhancing the structural stability of the electrode. This dual functionality—capacity contribution from low-valence Si and buffering from high-valence Si—highlights the advantage of the engineered Si_5_O structure synthesized via HEMM.

**Figure 4:**
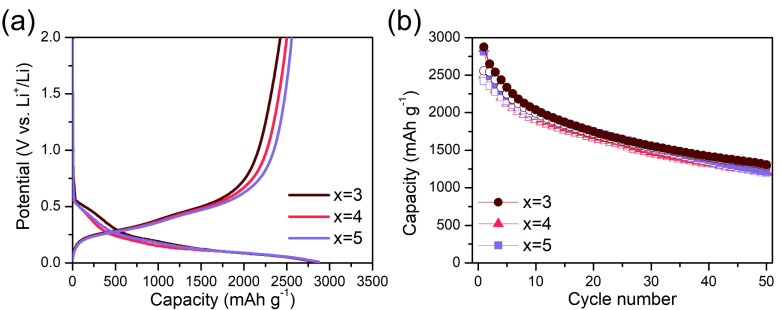
(**a**) Voltage profiles and (**b**) cycling performance of the Si_x_O composite electrodes (x = 3, 4, and 5).

Figure 4a shows the first-cycle voltage profiles of the Si_x_O (x = 3, 4, and 5) composite electrodes in Li half-cells at a constant current of 100 mA g^−1^. The reversible (charge) capacities for Si_3_O, Si_4_O, and Si_5_O were 2423, 2500, and 2558 mAh g^−1^, respectively, demonstrating that reversible capacity increases with higher Si content. The ICE values were 86%, 88%, and 89%, respectively, also increasing with Si content. This trend is expected, as Si actively alloys with Li in a reversible process, whereas SiO*_x_* suboxide undergoes irreversible reactions with Li.

Figure 4b presents the cycling performance of the Si_x_O (x = 3, 4, and 5) composite electrodes. While a higher Si ratio typically leads to increased reversible capacity, it also tends to degrade cycle performance due to the significant volume expansion of Si during cycling. However, despite the increasing Si ratio, the capacity after 50 cycles remains similar, at 1195, 1204, and 1294 mAh g^−1^, respectively. The capacity retention rates, relative to the first-cycle reversible capacity, are 43%, 43%, and 45%, respectively. Notably, despite the increase in Si content, the capacity retention rates exhibit minimal variation. This suggests that even a small amount of oxygen can stabilize the cycling performance of Si oxide electrodes with high Si content. Consequently, increasing the Si ratio in Si oxides offers a balance between high capacity and stable cycling performance while also achieving a relatively high ICE (e.g., 89% for Si_5_O).

**Figure 5:**
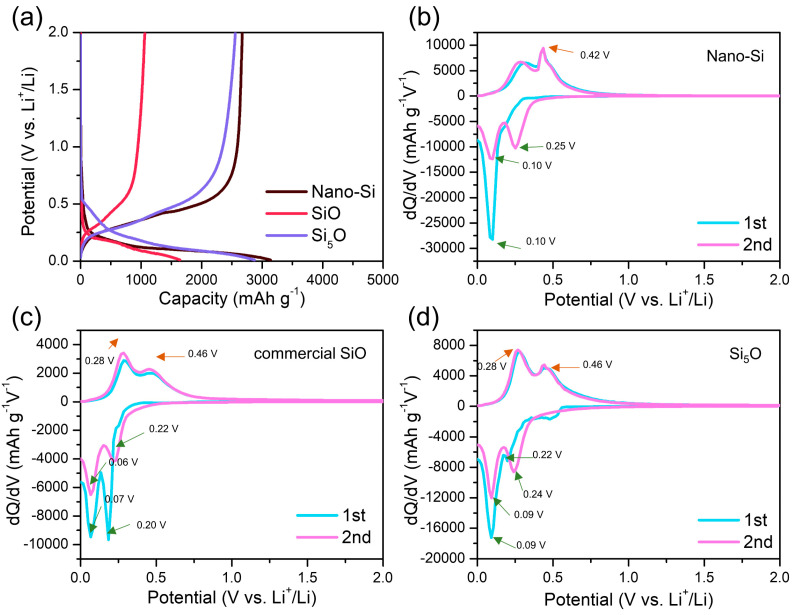
(**a**) Voltage profiles of nano-Si, commercial SiO, and Si_5_O; DCPs of (**b**) nano-Si, (**c**) commercial SiO, and (**d**) Si_5_O composite electrodes.

The voltage profiles and differential capacity plots (DCPs) of the precursor materials (nano-Si and commercial SiO) and the synthesized Si_5_O composite electrodes are shown in Figure 5. As observed in Figure 5a, the reversible capacities of nano-Si, SiO, and Si_5_O were 2653, 1061, and 2558 mAh g^−1^, respectively, with ICE values of 85%, 65%, and 89%. Notably, the synthesized Si_5_O electrode exhibits a higher ICE than nano-Si. This is likely because nano-Si has a significantly larger surface area, leading to a higher concentration of surface silicon oxide (Si–O–Si) and silanol (Si–OH) bonds [35]. Additionally, nano-Si undergoes significant volume expansion during the first lithiation (discharge), which results in incomplete delithiation during the first charge cycle. As previously shown in SEM and HR-TEM images, the synthesized Si_5_O composite consists of evenly distributed Si nanocrystallites embedded in a SiO*_x_* matrix. This structure effectively buffers the mechanical stress caused by volume changes, contributing to improved cycling stability. The presence of a surrounding SiO*_x_* matrix and the reduced particle size of Si_5_O likely enhance its ICE compared to nano-Si [35].

To gain deeper insights, DCPs were analyzed, where negative dQ/dV values indicate Li^+^ insertion and positive values correspond to Li^+^ extraction. Figure 5b presents the DCPs of the nano-Si electrode for the first two cycles. During the first lithiation, a peak at 0.10 V (vs. Li^+^/Li) appears, corresponding to the formation of crystalline Li_15_Si_4_. During delithiation, a sharp peak at 0.42 V indicates the dealloying of Li_15_Si_4_ [36,37]. Previous studies suggest that crystalline Si becomes amorphous after the first cycle, adopting the characteristics of an amorphous Si electrode in subsequent cycles.

Figure 5c shows the DCPs of the commercial SiO electrode for the first two cycles. In the first lithiation, peaks appear at 0.20 and 0.07 V (vs. Li^+^/Li), while in the second lithiation, smaller peaks emerge at 0.22 and 0.06 V. The reduced peak intensities in the second cycle suggest the occurrence of both reversible and irreversible reactions. The peaks around 0.20 and 0.22 V are associated with the formation of Li_2_O and Li_4_SiO_4_ phases, primarily from irreversible reactions. During the first and second delithiation, broad peaks at 0.28 and 0.46 V indicate reversible reactions, closely resembling the electrochemical behavior of amorphous Si [38,39].

Figure 5d presents the DCPs of the synthesized Si_5_O electrode for the first two cycles. In the first lithiation, peaks appear at 0.50, 0.22, and 0.09 V (vs. Li^+^/Li), while in the second lithiation, peaks shift to 0.24 and 0.09 V. The 0.22 V peak corresponds to the formation of Li_2_O and Li_4_SiO_4_ phases, while the 0.24 V peak in the second cycle is attributed to the lithiation of amorphous Si. The 0.09 V peak, associated with the formation of the Li_15_Si_4_ phase, confirms that crystalline Si in the Si_5_O composite reacts with Li. During delithiation, peaks at 0.27 and 0.44 V indicate reversible reactions, with the 0.44 V peak reflecting the behavior of amorphous Si [40]. These DCP analyses confirm that the Si_5_O composite, formed through the reaction of nano-Si and SiO during milling, consists of both crystalline Si and SiO*_x_* phases. The presence of a small amount of oxygen results in the formation of irreversible phases that help mitigate volume expansion in Si.

Figure S5 compares the cycling performance of the synthesized Si_5_O and precursor nano-Si electrodes. The capacity of nano-Si declines sharply within 20 cycles, whereas the Si_5_O electrode exhibits a more gradual capacity fade. This improved stability is attributed to the uniform distribution of Si nanocrystallites within the SiO*_x_* matrix, which buffers the volume changes in Si during cycling. The SiO*_x_* matrix plays a crucial role in enhancing structural stability, even with a high Si content in the Si_5_O composite electrodes.

**Figure 6:**
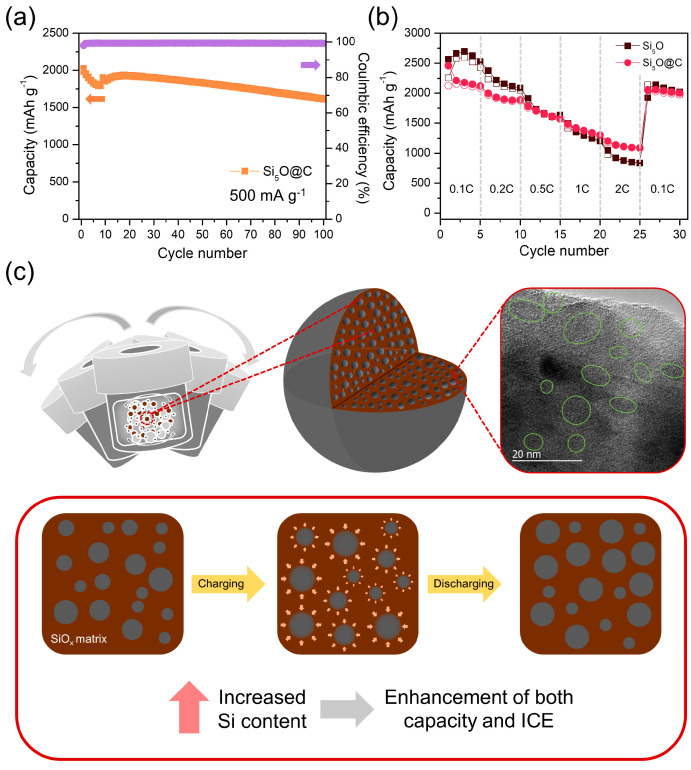
(**a**) Cycling performance of the Si_5_O@C composite electrode and (**b**) rate performance of the Si_5_O and Si_5_O@C composite electrodes; (**c**) schematic illustration of the composite electrode during Li insertion and extraction cycling.

To further enhance the electrochemical performance of the Si_5_O electrode, a carbon coating was applied via the carbonization of a naphthalene precursor [41]. The carbon incorporation was confirmed in the Raman spectra (Figure S6). As shown in Figure S7, the carbon-coated Si_5_O (Si_5_O@C) composite exhibits a uniform amorphous carbon layer with a thickness of a few nanometers. This is further supported by EDS mapping, where the C signal overlays the Si signal, indicating conformal carbon coverage on the active material. This carbon layer not only improves the poor electrical conductivity of Si_5_O but also acts as a buffer to accommodate volume changes. Figure 6a presents the cycling performance of the carbon-coated Si_5_O (Si_5_O@C) composite electrode at a current density of 500 mA g^−1^. The initial reversible capacity was 1984 mAh g^−1^, and after 100 cycles, it remained at 1609 mAh g^−1^. The Si_5_O@C electrode also exhibited improved cycling stability, with a capacity retention of 81% at high current densities, attributed to the protective effects of the carbon coating. During the initial 10–20 cycles, the capacity of the composite electrode gradually increased, which is attributed to the activation of electrochemically accessible Si and the stabilization of the SEI layer.

Figure 6b compares the rate performance of the pristine Si_5_O and carbon-coated Si_5_O (Si_5_O@C) electrodes in Li-ion half-cell tests. Both electrodes were tested at current densities ranging from 0.1 to 2 A g^−1^, with their voltage profiles shown in Figure S8. As the C-rate increased, the capacity of Si_5_O declined sharply, whereas the Si_5_O@C electrode exhibited a more gradual decrease. Additionally, at a 2C rate, Si_5_O@C retained a significantly higher capacity than Si_5_O, confirming that carbon incorporation effectively enhances rate performance. The schematic illustration of the carbon-coated, nano-Si-embedded Si_5_O composite materials during Li insertion and extraction cycling is displayed in Figure 6c.

To further investigate the effect of carbon coating on rate capability, EIS measurements were conducted, and the Nyquist plots are shown in Figure S9. Each plot consists of two semicircles in the medium-frequency region and a sloped line in the low-frequency region. The intercept of the medium-frequency semicircle represents interfacial resistance, including the SEI (R_SEI_) and charge transfer resistance (R_ct_) [42]. Before cycling, the intercept values were 400 Ω for Si_5_O and 190 Ω for Si_5_O@C. After 30 cycles, the Si_5_O@C electrode exhibited a significantly lower intercept value (21 Ω) compared to Si_5_O (60 Ω), demonstrating that carbon incorporation effectively reduces resistance and enhances rate performance. Table S1 shows a comparison of electrochemical properties for SiO*_x_*-based anodes [43,44,45,46,47,48,49,50,51]. The synthesized Si_5_O@C composite achieves a well-balanced performance, combining a high ICE (86.5%), a large reversible capacity (2126 mAh g^−1^), and excellent cycling stability (81% after 100 cycles at 0.5 A g^−1^), underscoring its practical viability.

To evaluate the commercial viability of Si_5_O@C in LIBs, it was blended with commercial graphite to form a composite anode (Si_5_O@C/G). The Si_5_O@C/G anode was designed to achieve an initial reversible capacity of approximately 518 mAh g^−1^ using a graphite-to-Si_5_O@C weight ratio of 9:1, substantially exceeding the theoretical capacity of graphite (372 mAh g^−1^) (Figure S10a). A full cell incorporating the Si_5_O@C/G anode and the LiNi_0.8_Co_0.1_Mn_0.1_O_2_ (NCM 811) cathode was constructed to verify its practical feasibility. The voltage profiles of the NCM811–Li half-cell and the full cell are shown in Figure S10b and S10c, respectively. The cycling performance of the full cell exhibited excellent long-term cycling stability (Figure S10d), confirming the effectiveness of the Si_5_O@C/G composite anode.

## 4. Conclusions

This study demonstrated a simple and scalable high-energy milling approach to enhance both the reversible capacity and ICE of Si suboxide-based anode materials by increasing the Si content. A higher Si ratio resulted in notable improvements in reversible capacity and ICE, with minimal compromise in capacity retention. The synthesized materials were thoroughly characterized using XRD, Raman spectroscopy, XPS, HR-TEM, and EDS, confirming the homogeneous nanoscale distribution of Si crystallites within the amorphous SiO*_x_* matrix. This controlled microstructure effectively mitigates the volume expansion of Si during cycling, contributing to improved electrochemical stability. Importantly, the synthesis method used is low-cost, does not require complex processing steps, and allows for precise control over the Si:SiO*_x_* ratio, making it well-suited for practical applications. The composite electrodes exhibited a favorable balance of high capacity, ICE, cycling stability, and rate performance—characteristics desirable for commercial lithium-ion battery anodes when blended with carbon. Overall, this work presents an affordable and controllable strategy for developing high-performance Si-based anode materials that match the capacity of nano-Si while significantly extending cycle life. This approach provides a promising pathway toward the commercialization of high-capacity Li alloy anodes.

## Figures and Tables

**Table 1:** Abundance ratios for Si valence states of the samples in Si 2p XPS spectra.

Sample	Si^0^ (%)	Si^1+^ (%)	Si^2+^ (%)	Si^3+^ (%)	Si^4+^ (%)
Si_5_O	31.6	31.1	21.1	10.6	5.6
Si_4_O	29.1	28.1	20.9	12.9	9.0
Si_3_O	27.9	26.8	20.0	14.1	11.2
Commercial SiO	3.1	28.4	20.9	24.0	23.6
Nano Si	42.9	26.5	14.9	9.6	6.1

## Data Availability

The original contributions presented in this study are included in the article/Supplementary Material. Further inquiries can be directed to the corresponding author.

## Supplementary Materials

The following are available online at: https://www.mdpi.com/article/10.3390/ma18143272/s1, Figure S1: XRD patterns of SixO composites (x = 7 and 10), Figure S2: FT-IR spectra of Nano-Si, SiO, and SixO (x = 3, 4, and 5), Figure S3: FE-SEM images of (a) nano-Si, (b) commercial SiO, and (c) Si_5_O, material milled for 24 h, Figure S4: HR-TEM images of (a) nano-Si and (b) Si_5_O,, Figure S5: Cycling performance of nano-Si and Si_5_O, electrodes, Figure S6: Raman spectra of the Si_5_O, and Si_5_O,@C composite materials, Figure S7: (a, b) Low-magnification TEM images, (c) EDS elemental mapping results, (d) high-magnification TEM images, and (e, f) HR-TEM images (inset: FFT pattern) of the carbon-coated Si_5_O, composites, Figure S8: Voltage profiles of Si_5_O,@C composite electrode at various rates, Figure S9: (a) Equivalent circuit model and EIS Nyquist plots of the (b) Si_5_O, and (c) Si_5_O,@C composite electrodes before and after 30 cycles, Figure S10: Voltage profiles of (a) the Si_5_O,@C/G anode, the LiNi_0.8_Co_0.1_Mn_0.1_O_2_ (NCM 811) cathode, and (c) full cell with both electrodes, as well as (d) the cycling performance of the full cell, Table S1: Comparison of the electrochemical properties of various SiO*_x_*-based anodes. Reference [52] is cited in the supplementary materials.
