# Supplementary material for: SiOx-Based Anode Materials with High Si Content Achieved Through Uniform Nano-Si Dispersion for Li-Ion Batteries
Source: Materials (Basel). 2025 Jul 11;18(14):3272. doi: 10.3390/ma18143272 (PMC12300280; doi:10.3390/ma18143272)
Supplement: Supplementary file 1 [file materials-18-03272-s001.zip › materials-3716135-supplementary.pdf]

Supplementary Materials

SiO_x-based Anode Materials with High Si Content Achieved through Uniform Nano-Si Dispersion for Li-ion Batteries

Seunghyeok Jang and Jae-Hun Kim*

School of Materials Science and Engineering, Kookmin University, Seoul 02707, Republic of Korea

*Corresponding author.

E-mail address: jaehunkim@kookmin.ac.kr (J.-H. Kim)

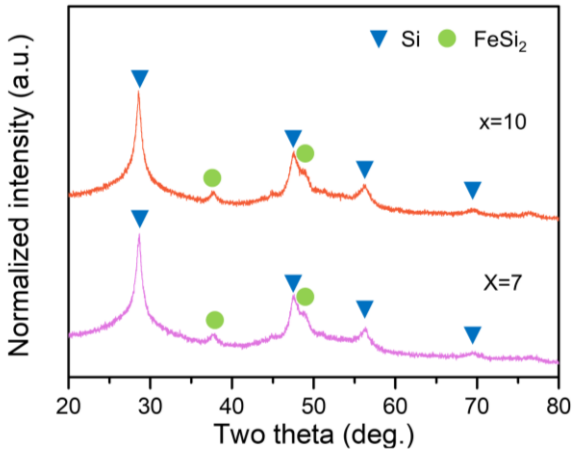

Figure S1. XRD patterns of Si_xO composites (x = 7 and 10).

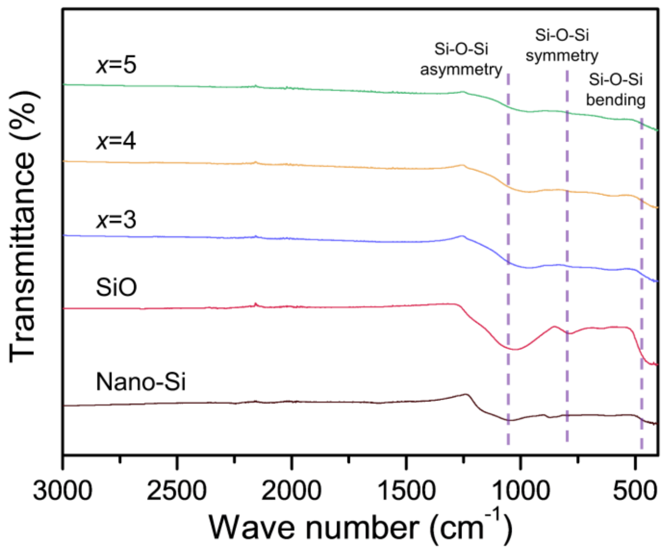

Figure S2. FT-IR spectra of Nano-Si, SiO, and Si_xO (x = 3, 4, and 5)

A decrease in the intensity of the Si–O–Si asymmetric stretching peak (~1100 cm^{-1}) is observed with increasing Si content. Additionally, the peak shifts to lower wavenumbers, indicating a reduction in the Si–O bond force constant due to decreased oxidation. These findings further support the compositional transition from oxide-rich to Si-rich [S1].

[S1] Helmiyati, H.; Suci, R.P. Nanocomposite of Cellulose-ZnO/SiO_2 as Catalyst Biodiesel Methyl Ester from Virgin Coconut Oil. *AIP Conf. Proc.* 2019, 2168, 020007.

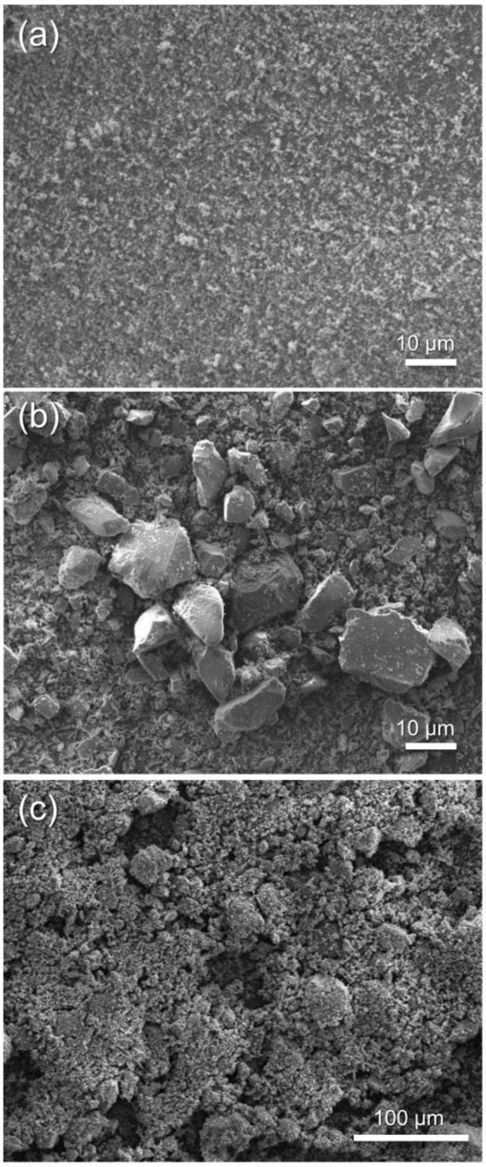

Figure S3. FE-SEM images of (a) nano-Si, (b) commercial SiO, and (c) Si_5O material milled for 24 h.

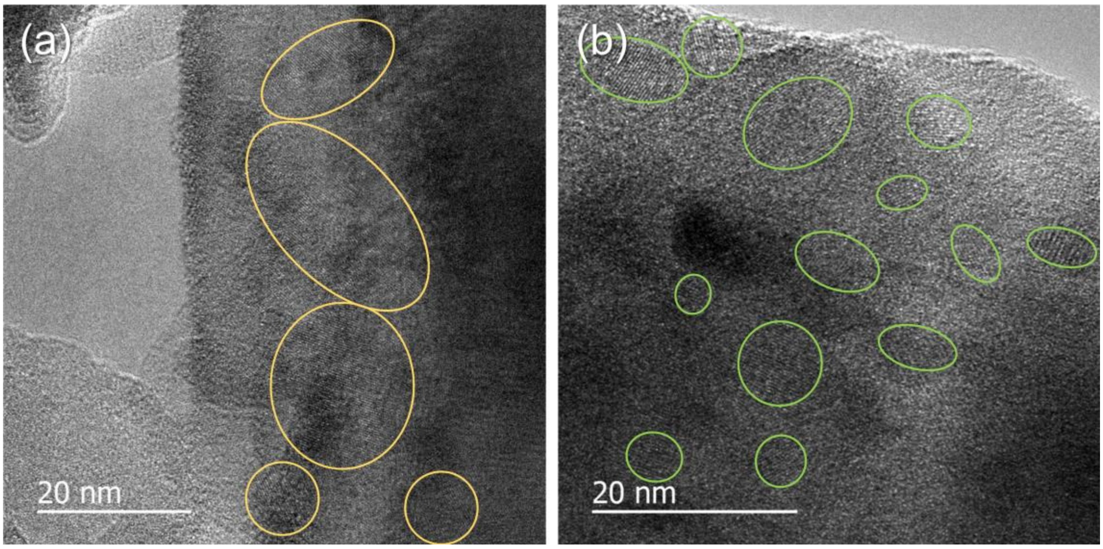

Figure S4. HR-TEM images of (a) nano-Si and (b) Si_5O.

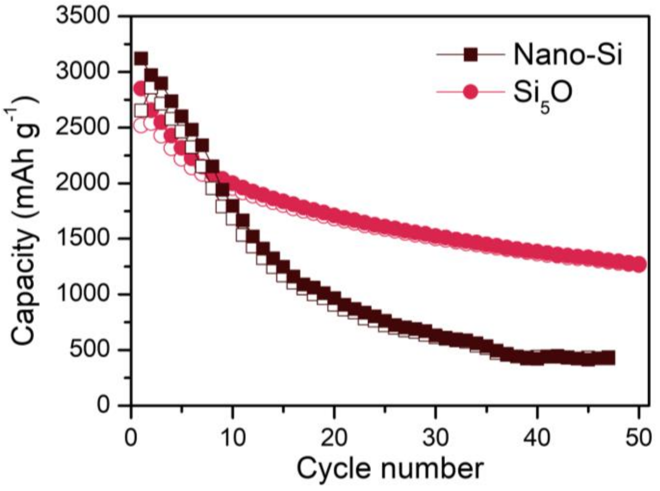

Figure S5. Cycling performance of nano-Si and Si_5O electrodes.

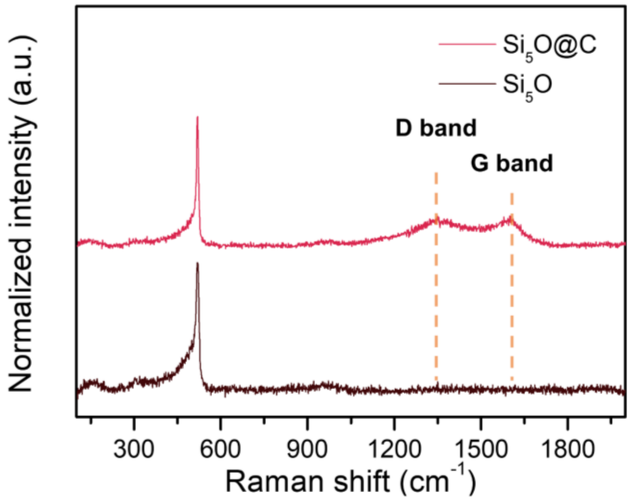

Figure S6. Raman spectra of the Si_5O and Si_5O@C composite materials.

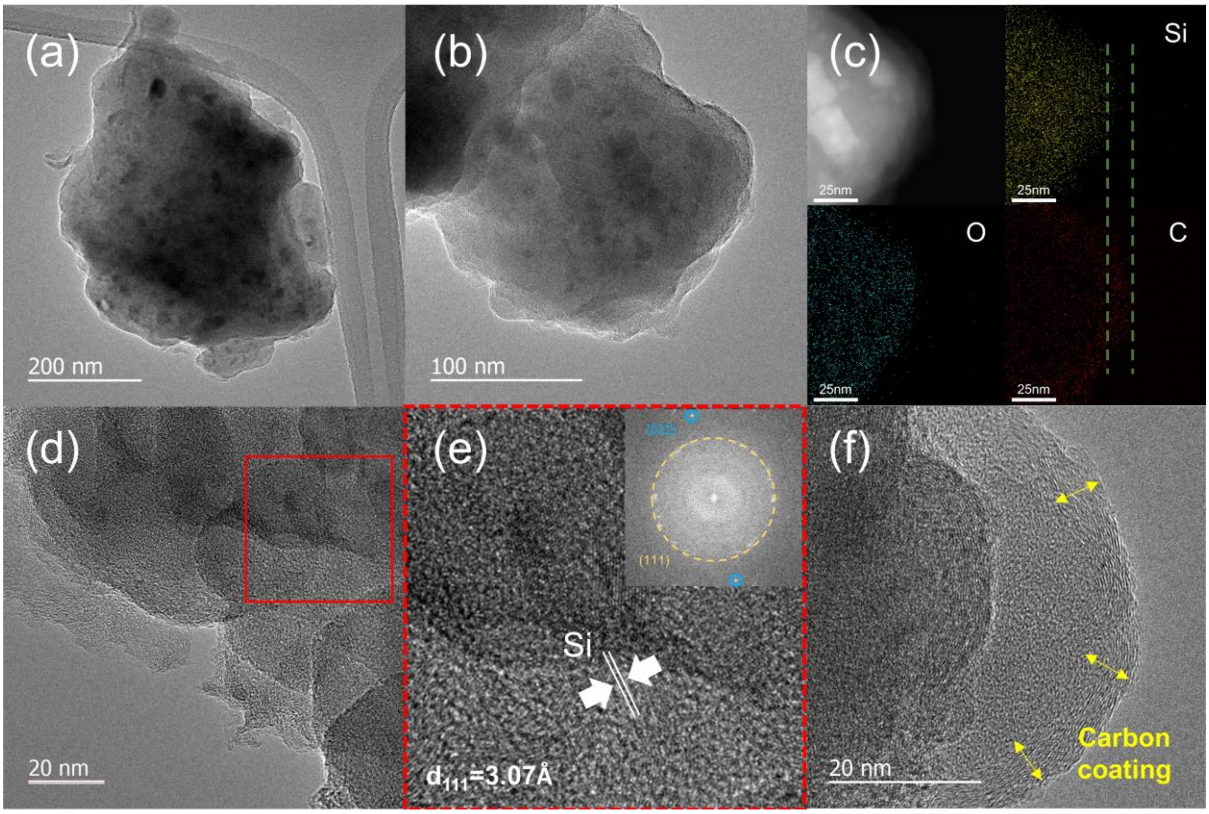

Figure S7. (a, b) Low-magnification TEM images, (c) EDS elemental mapping results, (d) high-magnification TEM images, and (e, f) HR-TEM images (inset: FFT pattern) of the carbon-coated Si_5O composites.

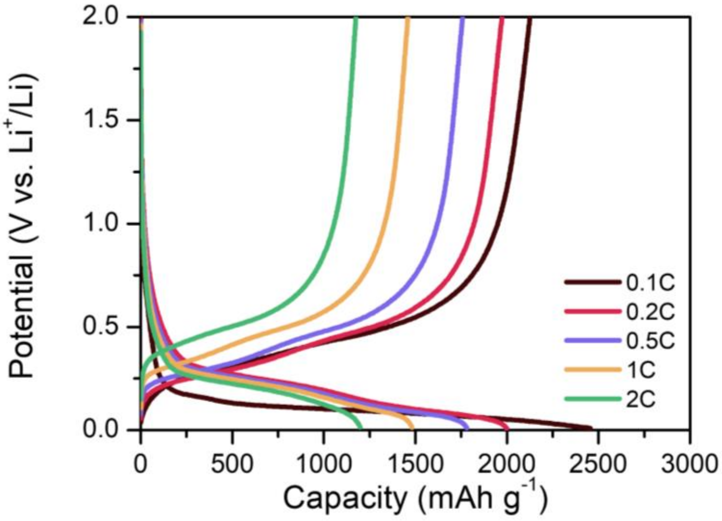

Figure S8. Voltage profiles of Si_5O@C composite electrode at various rates.

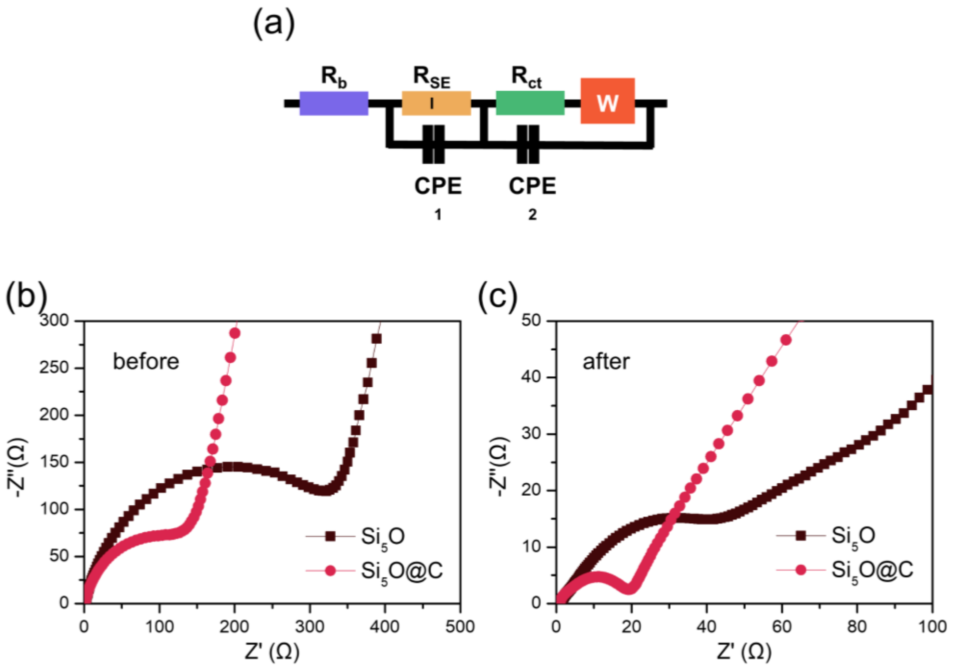

Figure S9. (a) Equivalent circuit model and EIS Nyquist plots of the (b) Si_5O and (c) Si_5O@C composite electrodes before and after 30 cycles.

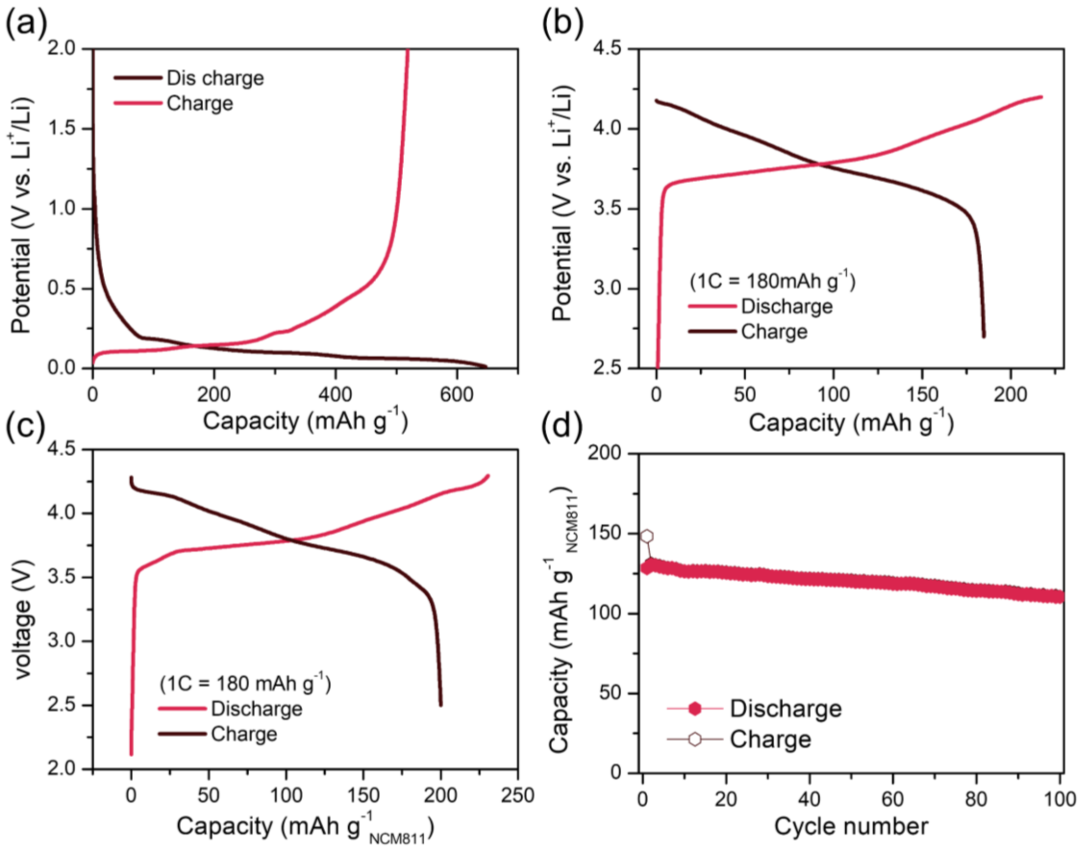

Figure S10. Voltage profiles of (a) the Si_5O@C/G anode, the $LiNi_{0.8}Co_{0.1}Mn_{0.1}O_2$ (NCM811) cathode, and (c) full cell with both electrodes; (d) cycling performance of the full cell.

Table S1. Comparison of the electrochemical properties of various SiO_x-based anodes.

Electrodes	ICE	Capacity [mAh g^{-1}]/ Current	Cycles/ Current	Capacity after cycles [mAh g^{-1}]	Capacity retention	Refs.
Micron-SiO_x/C	79.1%	1524.0/0.2 A g^{-1}	100/0.2 A g^{-1}	1409.7/50th	92.5%	[43]
SiO_x@C	77.8%	941.7/1.0 A g^{-1}	100/1.0 A g^{-1}	N/A	80.0%	[44]
P/SiO_x/C	73.5%	1077.0/0.1 A g^{-1}	200/1.0 A g^{-1}	720.0/200th	N/A	[45]
Si@SiO_x/C	69.6%	1167.0/0.1 A g^{-1}	350/1.0 A g^{-1}	1049.0/200th	93.4%	[46]
Si@SiO_x@C	84.0%	1981.0/0.2 A g^{-1}	300/1.0 A g^{-1}	N/A	65.0%	[47]
Si@SiO_x@SiC/C	83.4%	1195.0/0.1 A g^{-1}	325/0.2 A g^{-1}	614.0/325th	58.6%	[48]
SiO_x@C	54.0%	851.0/0.1 A g^{-1}	400/1.0 A g^{-1}	506.9/400th	93.0%	[49]
MnO-SiO_x@C	77.2%	857.8/0.1 A g^{-1}	100//0.3 A g^{-1}	724.0/100th	≈98.9%	[50]
p-Si@C	84%	624/0.1 A g^{-1}	120/0.1 A g^{-1}	624/120th	98%	[51]
This work [Si_5O@C]	86.5%	2125.5/0.1 g^{-1}	100/0.5 A g^{-1}	1608.6/100th	81%	
